# HIV Treatment as Prevention: Principles of Good HIV Epidemiology Modelling for Public Health Decision-Making in All Modes of Prevention and Evaluation

**DOI:** 10.1371/journal.pmed.1001239

**Published:** 2012-07-10

**Authors:** Wim Delva, David P. Wilson, Laith Abu-Raddad, Marelize Gorgens, David Wilson, Timothy B. Hallett, Alex Welte

**Affiliations:** 1 South African Department of Science and Technology/National Research Foundation Centre for Excellence in Epidemiological Modelling and Analysis, University of Stellenbosch, Stellenbosch, South Africa; 2 Kirby Institute, University of New South Wales, Sydney, New South Wales, Australia; 3 Infectious Disease Epidemiology Group, Weill Cornell Medical College—Qatar, Cornell University, Qatar Foundation—Education City, Doha, Qatar; 4 Department of Public Health, Weill Cornell Medical College, Cornell University, New York, New York, United States of America; 5 Vaccine and Infectious Disease Division, Fred Hutchinson Cancer Research Center, Seattle, Washington, United States of America; 6 Global HIV/AIDS Program, The World Bank, Washington, District of Columbia, United States of America; 7 Department of Infectious Disease Epidemiology, Imperial College London, London, United Kingdom; Duke University Medical Center, United States of America

## Abstract

Public health responses to HIV epidemics have long relied on epidemiological modelling analyses to help prospectively project and retrospectively estimate the impact, cost-effectiveness, affordability, and investment returns of interventions, and to help plan the design of evaluations. But translating model output into policy decisions and implementation on the ground is challenged by the differences in background and expectations of modellers and decision-makers. As part of the *PLoS Medicine* Collection “Investigating the Impact of Treatment on New HIV Infections”—which focuses on the contribution of modelling to current issues in HIV prevention—we present here principles of “best practice” for the construction, reporting, and interpretation of HIV epidemiological models for public health decision-making on all aspects of HIV. Aimed at both those who conduct modelling research and those who use modelling results, we hope that the principles described here will become a shared resource that facilitates constructive discussions about the policy implications that emerge from HIV epidemiology modelling results, and that promotes joint understanding between modellers and decision-makers about when modelling is useful as a tool in quantifying HIV epidemiological outcomes and improving prevention programming.

## Introduction

In almost all areas of public health, mathematical models are used to provide quantification and insight that can inform decision-making. Epidemiological data can be collected about individuals, and clinical trials can measure individual-level effects in a selected study population (often under best-case circumstances), but public health decision-making requires an understanding of the dynamics of disease across a population under a variety of conditions. Mathematical modelling aims to unite knowledge and assumptions about behavioural dynamics, biology, costs, and constraints to generate estimates of impact and cost-effectiveness, and recommendations for resource allocation.

Models are especially useful in the case of infectious diseases, where they can estimate temporal changes in disease burden and treatment needs, and so underpin projections of the counterfactuals in some quasi-experimental impact evaluation designs, and power calculations for prospective experimental study designs. These are important applications, especially in contexts where empirical data are not available. Thus, models have increased in prominence over the last several years, including in establishing optimal responses to emerging pathogens [1] and influenza pandemics [2], examining the conditions for polio eradication [3] and malaria control [4], and making a case for restructuring investment in HIV programs [5],[6].

Investigators from many different disciplines generate models, and the techniques and presentation formats employed have tended to follow a corresponding diverse set of conventions and presumptions. Meanwhile, those who rely on modelling output have highly varied needs and expectations from epidemiological modelling analyses. It is not uncommon for different models addressing very similar questions to produce—or appear to produce—widely different estimates [7], and thus a model's validity and ability to inform an important public health decision can be questioned.

Therefore, there is a need for constructive dialogue between “producers” and “consumers” of modelling results about a model's assumptions and structure, the policy implications of the results, and what further empirical and modelling studies should be planned. The World Bank Global HIV/AIDS Program, as a funder, coordinator, and evaluator of HIV prevention efforts, has become increasingly reliant on mathematical modelling and has initiated a modelling guidelines development process through its Prevention Science and Mathematical Modelling Reference Group, a panel of experts in HIV prevention, and modelling relating to HIV prevention, created and convened by the World Bank on the basis of individuals' publication records and institutional roles. In consultation with the reference group and other HIV modelling experts, we have developed a set of principles for the construction, reporting, and interpretation of HIV epidemiological models for public health decision-making on all aspects of HIV.

## Development and Scope of the Recommendations

The nine principles, discussed below and summarised in Table 1, were initially identified during a number of discussions within the context of collaboration amongst the authors, within the HIV Modelling Consortium and the World Bank modelling guidelines production process. Written input on the nine principles was solicited from a wider group of modellers, including former and current collaborators. This was followed by a three-day work retreat of five of the authors, during which a first draft was produced, based on the authors' experience and other researchers' responses to the proposed core principles. The resulting draft was presented to a meeting of the World Bank Prevention Science and Mathematical Modelling Reference Group, and revised in light of feedback received.

**Table 1:** Summary of principles of good HIV epidemiology modelling.

Principle	Model Producer Considerations	Model Consumer Considerations
**Clear rationale, scope, and objectives**	Are the rationale, scope, and objectives clearly stated?	Are the rationale, scope, and objectives understood?
	Is there a statement about why epidemiological modelling is appropriate for this problem?	Is epidemiological modelling appropriate for this problem?
**Explicit model structure and key features**	Is the model structure completely described, such that all analyses can be reproduced?	Is the model presented comprehensively, such that the inclusion/exclusion of any particular assumption or feature can be identified?
	Is there a description of key model features?	Is the justification for model structure/key assumptions reasonable, considering the primary rationale, scope, and objectives of the study?
	Has a justification for the model structure been provided?	
**Well-defined and justified model parameters**	Is there an understandable and complete listing of the model parameters, their values, and their justification?	Are the implicit inputs upon which the model predictions are made understood, and are they satisfactorily justified?
**Alignment of model output with data**	Are the model fitting, calibration, and validation approaches with respect to relevant data defined and justified?	Does the model produce, or fail to produce, outputs that can be compared to real world data, and does the model output reflect realistic conditions?
		Does the comparison with real world data increase confidence in the suitability of the model for the current enquiry?
**Clear presentation of results, including uncertainty in estimates**	Have the uncertainties been captured for all relevant factors included in the model?	Have the uncertainties been captured for all relevant factors included in the model?
	Is the key result of the study robust to that uncertainty?	Are the results sufficiently robust for confident decision-making, or is further analysis or data collection required?
	Are specific recommendations for new data analyses/collections appropriate?	
**Exploration of model limitations**	Are sufficient details provided about limitations of the study, specifically about model structure, parameterization, and application/generalisability?	Are the limitations of the model and its findings clearly understood, including the limits of applicability and generalisability?
		Considering the strength of the evidence, how are the model findings relevant for informing public health decision-making?
**Contextualisation with other modelling studies**	Have relevant previous studies been referenced and differences/similarities discussed?	Is there an understanding of the overarching conclusion(s) from modelling studies on the topic?
	Is it clearly specified whether a new result versus a confirmation/contradiction of a previous result is presented?	Are the general reasons (assumptions or underlying real world conditions) for why models differ in their conclusions understood?
**Application of epidemiological modelling to health economic analyses**	Where relevant, are understandable and appropriate estimates of epidemiological impact provided, such that health economic inferences can be made?	Can the model-based estimates be used to infer cost-effectiveness measures of relevant interventions or be extended to health economics?
		Is the degree of uncertainty in estimates relevant to cost-effectiveness understood, particularly with respect to the sensitivity of key parameters?
**Clear language**	Are model scenarios described in clear formal terms (separate from interpretations about reality) that facilitate technical understanding and evaluation?	Are there clear explanations of intended correspondences between inputs used in the model and key real world conditions such as epidemiological conditions, policy, and programmes?

Our focus complements more general reviews of modelling [8]–[10] and technical content in modelling textbooks [11],[12]. The recommendations are intended for all HIV public health practitioners who rely on modelling research to make policy decisions, as well as those conducting the modelling research itself. They are not intended to be prescriptive, and hence should not be seen as a normative checklist against which to score the quality or validity of modelling studies. For instance, where mathematical models are used to construct a simple *conceptual* framework of behavioural, clinical, virological, and/or epidemiological dynamics, rather than to conduct research for public health decision-making, some of the recommendations in this article may not be applicable.

## Principle 1: Clear Rationale, Scope, and Objectives

As in any scientific report, the rationale, scope, and objectives of a modelling study should be clearly stated. The reporting of a modelling study should include an explicit explanation for why epidemiological modelling, rather than another study design (e.g., systematic review, meta-analysis, quasi-experimental design, or a randomized controlled trial), is appropriate for the problem, the exact questions the work seeks to address, and the readership for which it is intended. This statement of rationale, scope, and objectives provides the criteria against which all modelling decisions should be judged, assists in framing the interpretation of the work, and should be referred to at key points throughout the write-up, to maintain the alignment of aims, model, results, and interpretation. Examples might be: “We aimed to generate estimates for the cost of rolling out a male circumcision programme in South Africa so that stakeholders can compare these costs against those of other possible interventions, and use the comparison to inform decisions about allocation of funding”; “We aimed to explore the extent to which HIV incidence rates can be influenced by changes in condom use among sex workers and their clients under different assumptions about sexual mixing patterns in concentrated HIV epidemics, so that recommendations can be made for data collection during the implementation of a condom distribution campaign”.

For studies that aim to estimate the potential population-level impact of a given biomedical intervention, there are differences in emphasis in their purpose that should be clear from the outset and throughout the presented work. An important distinction is between investigation of the potential benefits of a hypothetical biomedical intervention that is currently in development but has unknown efficacy, and an intervention that has a proven efficacy, such as from a trial setting. Typically, the purpose of the first type of study is to estimate the population-level effectiveness of the hypothesized intervention and to identify key properties the intervention would need to have to be effective (such as for vaccines [13]–[15], microbicides [16],[17], and chemoprophylaxis [18]), whereas the purpose of the second type of study is to guide targeted implementation of the intervention in real populations (such as deciding which populations should be circumcised first [19], or prioritised for treatment as prevention [20]). Another distinct form of modelling study is where an assessment is generated for the epidemiological impact of a previously implemented public health program [21].

## Principle 2: Explicit Model Structure and Key Features

The model chosen for the analysis should be described completely and clearly (commonly in the form of an online technical appendix, ideally with the model's computer code made available), so that other investigators can reproduce its findings and projections. Justification for the choice of model (individual- versus population-based, stochastic versus deterministic, linear versus nonlinear) should be provided, along with a description of the model's structure and key features, with cross-references to the scope and objectives. A flow diagram, representing how individuals or subpopulations transition through the different demographic, behavioural, or clinical states in the model can be an excellent way to communicate the model's main structure.

The model structure, and the consequent key demographic, behavioural, biological, clinical, and epidemiological factors represented or omitted by the model, may affect the interpretation of the results. Certain biological or behavioural features of HIV transmission, prevention, and treatment may be at the core of the issue addressed by the model, and cannot be omitted. However, additional features that are irrelevant to the primary objectives of the analysis may obscure the main conclusions or may open unnecessary debate about the validity of parameter values that are not essential to interpretation of the model output [8]. Judging which features fall into which category may be informed by earlier research or explicit investigation, but is more commonly based on assumptions, which should at least be clearly stated. Furthermore, a mathematical model need not require an examination at all scales (e.g., within host, individual level, sexual network level, and population level); rather, scales to be included should be dictated by the objectives of the study (e.g., some models focus on within-host processes and thus must include the interaction between virus and immune cells, but models that focus on between-host transmission may not require detail at this scale). In general, the strength of the model should not be judged merely by the level of model detail and whether or not particular factors are included. Rather, the appropriateness of model detail and factors taken into account by the model should be assessed within the context of the scope and objectives.

Discussion of how the model structure could have influenced the results should always be included. Examples of formal evaluations of differently structured models addressing similar research questions but reaching different conclusions can be found in various branches within the infectious disease modelling field, e.g., in the modelling of chlamydia [22], influenza [23], and HIV epidemics [24]. It is often not feasible, in one article or within one modelling research group, to explore large differences in model structure, such as between deterministic population-based versus individual-based models. However, where possible, comparison between models is highly encouraged. For example, Johnson et al. [25] used two models in the same study to assess the impact of antiretroviral therapy (ART) and condom usage on HIV epidemics in South Africa, and Eaton et al. [7] discuss the implications of alternative model structures for estimating the potential impact of early initiation of ART on HIV incidence in hyperendemic settings. Such formal evaluations foster discussions of the reasons behind discrepancies in model predictions, and either pave the way for a consensus statement about the findings and conclusions that are most certain, or highlight key issues for further scientific enquiry.

## Principle 3: Well-Defined and Justified Model Parameters

Another set of assumptions in a model concerns the values that are given to the parameters. Examples of parameters include the probability of HIV transmission per sex act for an individual on ART, the fraction of patients still alive and on ART three years after ART initiation, and the annual population growth rate. It is essential for any modelling study to include a transparent listing of all model parameters, providing the following for each parameter: the name of the parameter; the mathematical symbol of the parameter (if appropriate); the meaning of the parameter in plain language; the value(s) assigned to the parameter (a point estimate and range/confidence interval as appropriate); and a contextual justification for used values, with references for the origins of the model parameter(s), and any relevant caveats (particularly important if more than one value for the model parameter exists or if the parameter is fit in the model or is derived from another modelling analysis).

This notion of justifying or formally “fitting” individual parameters—or a model in its entirety—to data covers many possibilities. As these also do not lie on a clear continuum from “rough heuristic/qualitative” to “formally rigorous and unbiased”, some ad hoc critical evaluation is appropriate for the most important inputs into any model. All model fitting relies on the notion of the likelihood of observing a set of data. There are then various possible approaches to (1) maximising the likelihood, i.e., selecting the particular model within which the data are most consistent, or (2) performing a sensitivity analysis, i.e., identifying ranges of model parameters that are consistent with the data and determining the relative importance of each model parameter. Note that the “likelihood function” itself can capture multiple sources of randomness, such as the usually unavoidable incompleteness of sampling and random effects in population processes themselves.

Some parameters, such as the mother-to-child HIV transmission rate under a particular care regimen, can be more or less directly “measured” in an appropriate (typically randomized) study, using observation and standard robust biostatistical methods, but there may be subtle artefacts. For example, using logistic regression to identify the characteristics of individuals that are associated with an HIV infection or transmission event may be misleading in ways that are seldom systematically explored in routine application, beyond noting the potential for “residual confounding”. A particular shape for a relationship between a predictor (such as viral load or age) and an outcome (transmission) is implicitly assumed, although it may be inappropriate—age in particular may correlate strongly with health status, but not necessarily monotonically.

For parameters where it is very difficult to obtain direct measurements, e.g., to capture behavioural dynamics such as risk reduction in the face of risk perception, heuristic parametrization may indicate which parameter sets are plausible and which are clearly at odds with data: a heuristically sensible model and a formally fitted model should be clearly distinguished, with sensitivity analyses where applicable.

Often the most important assumptions concern those specifying a simulated intervention, and it is recommended that these be prominently and exhaustively listed. For instance, if the intervention of interest relates to a policy change in ART, specifying a “coverage” and “efficacy” may not be enough: assumptions about enrolment rates, adherence, and retention, as well as behavioural characteristics (e.g., risk reduction or compensation) and demographic impacts (e.g., reduced mortality rates and increased size of the HIV-positive population) [7] may need to be made explicit. These specifications should be documented over the time period of the model simulation, and, where relevant, for different substrata of the modelled population. If the work is specific to a country, then it is helpful to involve relevant stakeholders in the decisions taken about parameter values, and this process should be described. Such documentation also assists when modelling findings are subsequently used to inform decision-making in that setting [26],[27].

## Principle 4: Alignment of Model Output with Data

Here the emphasis shifts to assessing the alignment of output from a particular epidemiological scenario model to data. Understanding the modelled scenarios produced, and relating these to data by back-fitting them to a model, naturally forms an important component of the evaluation and application of any model. It is particularly important to indicate whether, and to what extent, input parameters were chosen to maximise the correspondence of outputs to data, or whether correspondences emerged naturally from choosing externally justified inputs. Demonstrating that a model can reproduce observed patterns provides a certain level of reassurance that the model is capturing the system appropriately, and where models cannot demonstrate this, extreme caution should be taken in interpreting results.

The most desirable situation is when a model that has been fitted to some data (a training set) produces output in close correspondence with additional data (a testing set). There are two primary caveats to this approach: (1) fitting a smooth model to slowly varying data and extrapolating a little may be “too easy”, and might indicate little about the suitability of the model, and (2) in key applications relevant to impact evaluation, asking the model to produce other independent data may be an unreasonable demand, tantamount to asking a model to predict future changes in the financial or political context. There may be deeper differences between the scenarios producing the training/testing datasets than can realistically be captured by a model—such as changes in treatment uptake or effects of improved treatment programmes on mortality.

While correspondence between models and data is reassuring and potentially useful—if not taken as absolute confirmation of the correctness of either model structure or parameter values—it is important to consider whether there are multiple ways to fit the data, and to realise that there may be scientific progress in a failure to fit data, either at all or without resorting to implausible values, ranges, or correlations of parameters. For example, simple (biological) models of ART cannot reproduce both the consistently strong reductions in patient viral loads and the inability to achieve viral eradication observed in the real world, without implausible “fine tuning” of individual subjects' treatment efficacy parameters into a narrow range. This situation diagnoses a model limitation, namely, the neglect of the fact that interactions between cells, drugs, and virions vary among compartments within the infected host.

The difficulties of “correctly” capturing a complex set of shifting context-defining processes impinge not only on the interpretation of correspondence between models and historical data, but also on the interpretation of the predictive component of scenarios. One useful application of modelling, when there are insufficient data to construct scenarios with conventional predictive credibility, is to pose questions such as what characteristic of a program would be required for certain goals to be achieved (e.g., what level of risk compensation, captured in a suitably clearly defined parameter, would be required to negate the risk reduction of a planned intervention).

## Principle 5: Clear Presentation of Results, Including Uncertainty in Estimates

The output of any modelling study needs to be presented clearly, using explicitly defined metrics and with any deviance in the interpretation between the model metric and the real world analogue explained. The many assumptions involving the structure of the model, the parameter estimates, and the data will all have uncertainties, and it is important to understand how these propagate to key model outputs. In some cases, uncertainty in a particular parameter will be benign—a result is reached irrespective of any credible assumption about that parameter—and this serves to increase confidence in the findings. In other cases, different credible values for a parameter (or model structure or interpretation of data) would lead to different conclusions, and this should be noted.

Uncertainties are best depicted as part of the modelling results presentation—either in tables or as part of the graphical output of the model. If sufficient information is available about inputs, computational techniques can manufacture a distribution for model outcomes, so that the main result can be given as a “credible interval”. In addition to uncertainty analyses, formal sensitivity analyses of the importance of each model parameter in influencing the variability in model outcomes can be useful for identifying items for further data collection or investigation (see [28]–[30] for examples in HIV modelling). Bayesian melding approaches have also been used recently, and have the advantage that they integrate uncertainty analyses with model fitting: good examples in HIV transmission modelling include work by Alkema et al. [31] and Johnson et al. [32].

## Principle 6: Exploration of Model Limitations

As Box and Draper [33] wrote, “Remember that all models are wrong; the practical question is how wrong do they have to be to not be useful”. It is necessary for modellers to provide a description of model limitations and for model consumers to appreciate the caveats and limitations of modelling studies when considering their results. Many limitations are due to the data that are available and used to parameterize modelling studies. Direct observation of some of the model parameters is often not feasible. This is especially true in the case of HIV, where transmission dynamics are dependent on sensitive and private aspects of human behaviour [34]. Modelling strategies address this challenge in part by fitting the model to data to yield estimates for the unknown parameters.

One thing that modellers may implicitly understand but that model consumers may not—and which therefore should always be made clear—is that capturing complex reality is not really the purpose of mathematical models. Practicality implies that one can never capture full dynamical structure, such as all conceivable population compartments, transition rules, or stochasticity. A mathematical model is a minimalist approach to representing the essential elements of reality that are necessary and sufficient for addressing a specific research question [35],[36]. Models are often applied to specific settings, and so transferability of the predictions to other settings may be limited. Just as the findings of clinical trials can be subject to multiple interpretations, modelling studies similarly may have multiple interpretations, and even more readily admit various choices in emphasis, of which only a few receive a full airing in the investigators' report.

Some of the limitations of modelling studies can be addressed by uncertainty or sensitivity analyses as discussed above [28],[37],[38]. Probably the least appreciated mode by which limitations in models are addressed is by a comparative assessment of models and their predictions, similar to systematic reviews and meta-analyses of datasets. Recent examples of this kind of process include the male circumcision modelling consensus paper [19], a special edition of *Vaccine* that examined the potential impact of a partially effective vaccine [13], and model comparisons of the impact of ART on prevention presented in another article in the July 2012 *PLoS Medicine* Collection, “Investigating the Impact of Treatment on New HIV Infections” [7].

## Principle 7: Contextualisation with Other Modelling Studies

It is common for multiple modelling groups to attempt to address similar research questions but with different modelling approaches: using models that have been designed to describe different populations, involve different model structures, and make different parameter assumptions. Apparently conflicting results in the modelling literature may consequently lead to greater confusion for the consumers of models or to distrust in the use of models for decision-making. Therefore, it is necessary that interpretations of results are contextualised with previous modelling findings relevant to the topic. It should be made clear whether a new result is being presented or whether study findings concur with previously published results.

Meanwhile, journal editors should recognise the value of works that rigorously confirm or draw together previous findings. Review papers that summarise the modelling literature on a specific topic are highly useful (see the recent special issue on HIV epidemic modelling in *Current Opinion in HIV and AIDS* [39]). Also, papers that aim to present meta-analyses of model results (e.g., [24]) should be encouraged, as well as papers that compare modelling results to quasi-experimental results. Of even greater utility for policy-makers is the formulation of consensus documents that summarise conclusions from numerous modelling studies, and provide general conclusions in a single voice from the modelling community; this has been done for evaluations of circumcision interventions [19] and HIV vaccines [13], and this *PLoS Medicine* Collection on HIV treatment as prevention aims to move the field in that direction as well, although there is clearly much more to do [7],[40].

## Principle 8: Application of Epidemiological Modelling to Health Economic Analyses

A public health policy or programme decision-maker generally desires to take actions that will have maximal impact whilst minimising the amount of money required to achieve the health outcomes—based, for example, on estimates of either the maximum impact that can be achieved for a given amount of money, or the money needed to achieve specific set levels of impact. Therefore, the cost-effectiveness, affordability, and returns on investments of interventions are among the most important considerations in their potential implementation. HIV epidemic modelling studies often attempt to estimate the population-level impact associated with changes in programme or policy conditions, and hence estimate the denominator (effectiveness) in the incremental cost-effectiveness ratio. Ideally, such models should be designed to produce outputs amenable to recycling into analyses of cost implications and estimates of primary epidemiological effects that are understandable and relevant to decision-makers, such as the number of incident infections or deaths averted, quality-adjusted life years gained, or disability-adjusted life years averted. Effective assessment of affordability and cost-effectiveness may require different time horizons than those chosen in epidemiological modelling analyses, hence additional simulations may be necessary prior to attaching costs, benefits, and utilities to epidemiological model outputs.

There are numerous good examples of modelling studies that have provided outputs that are relevant for use in health economic calculations or that have been integrated into cost-effectiveness analyses [41]–[44]. Guidelines have been developed for the production, submission, and review of health economic analyses for *BMJ* [45]; some of the principles presented in those guidelines align with those presented here. When modelling studies have the potential to be extended to health economic calculations, consideration of these health economic guidelines is encouraged.

## Principle 9: Clear Language

A particular challenge that arises when using models to evaluate the impact of interventions is a lack of clarity around the intervention itself. Such a lack of clarity minimises the usefulness of results for policy-makers in deciding which interventions to prioritise. While modellers are usually keenly aware of the technical details of the model, the interpretation of model features—both in the input and output phase—is prone to oversimplification by both modellers and readers. It can be convenient but misleading to present a correspondence in the real world between an actual policy choice and future events. For instance, a write-up should highlight that what is modelled is a reduction in the proportion of “unprotected sex acts”, which is not an intervention per se but could be the outcome of an intervention (e.g., an increase in condom distribution points or a targeted education campaign).

It is probably better to risk erring on the side of repetitiveness in efforts to keep focusing on precise model assumptions (qualitative and quantitative), and for consumers to process the model first on its own terms, before evaluating model scenarios in broad correspondence to reality and potential policy implications. At the same time, it is important that modellers use language that facilitates easy communication, without loss of precision and of key real world messages to consumers.

## Conclusion

The issue of using models in decision-making is especially important for the field of HIV prevention, which has now reached a critical point. Just as spending on HIV has levelled off or declined [46], there have been several significant scientific breakthroughs, including the finding that ART can substantially reduce the infectiousness of infected individuals [47]. This finding immediately conjures a multitude of questions that can be best examined through mathematical modelling. Examples of specific questions within the field would include (1) whether programs should reallocate funding to treatment in response to the new data [48], (2) the probability of drug resistance emerging as a threat to the therapeutic effectiveness of treatment [49], and (3) how the impact of real programs can be scientifically measured [50]. Further research questions are delineated in this *PLoS Medicine* Collection [40]. Our intention in compiling our recommendations is to help strengthen the support that mathematical models can provide in addressing such questions that are critical for setting research and intervention priorities for HIV.

**Key Points**

- Mathematical models are used to inform public health decision-making about many questions in the response to HIV epidemics, and here we present our recommendations for “best practices” for constructing, interpreting, and presenting such models.
- An overarching theme of our recommendations is that it is crucial for modellers to be explicit about the choices they make—about model structure, parameters, and model fitting and interpretation—and the reasoning behind their choices.
- Modellers need to make the limitations of their models clear, and model consumers (such as policy- and decision-makers) need to appreciate the caveats and limitations of modelling studies when considering their results.
- One of the least appreciated ways to address the limitations of models is through comparing the parameters, structure, and outputs of alternate models of the same processes.
- Especially useful are consensus documents that bring together conclusions from numerous modelling studies and summarise what researchers agree on and where uncertainty persists.

## Abbreviations

ART: antiretroviral therapy
